# A randomized feasibility study of the effect of ascorbic acid on post-angioplasty restenosis of hemodialysis vascular access (NCT03524846)

**DOI:** 10.1038/s41598-019-47583-w

**Published:** 2019-07-31

**Authors:** Chung-Wei Yang, Chih-Cheng Wu, Chien-Ming Luo, Shao-Yuan Chuang, Chiu-Hui Chen, Yung-Fang Shen, Der-Cheng Tarng

**Affiliations:** 1 Division of Nephrology, Department of Medicine, National Taiwan University Hospital, Hsinchu Branch, Hsinchu, Taiwan; 2 Cardiovascular Center, National Taiwan University Hospital, Hsinchu Branch, Hsinchu, Taiwan; 3 College of Medicine, National Taiwan University Hospital, Taipei, Taiwan; 4 Institute of Biomedical Engineering, National Tsing-Hwa University, Hsinchu, Taiwan; 5 Institute of Cellular and System Medicine, National Health Research Institute, Miaoli, Taiwan; 6 Division of Cardiovascular Surgery, Department of Surgery, National Taiwan University Hospital, Hsinchu Branch, Hsinchu, Taiwan; 7 Division of Preventive Medicine and Health Services Research, Institute of Population Health Sciences, National Health Research Institutes, Miaoli, Taiwan; 8 Department of Nursing, National Taiwan University Hospital, Hsinchu Branch, Hsinchu, Taiwan; 9 Department and Institute of Physiology, National Yang-Ming University, Taipei, Taiwan

**Keywords:** Outcomes research, Haemodialysis

## Abstract

Restenosis remains a significant problem after angioplasty of hemodialysis vascular access. Both experimental and clinical studies have shown a protective effect of antioxidants against post-angioplasty restenosis. A prospective, randomized, feasibility study was conducted to investigate the effect of ascorbic acid to prevent restenosis. Ninety-three hemodialysis patients were randomized into three groups after angioplasty: placebo (n = 31), 300 mg ascorbic acid (n = 31), and 600 mg ascorbic acid (n = 31), treated intravenously 3 times per week for 3 months. Eighty-nine completed the clinical follow-up, and 81 had angiographic follow-up. In the angiographic follow-up, the mean (stand deviation) late loss of luminal diameter for the placebo, 300 mg, and 600 mg groups were 3.15 (1.68) mm, 2.52 (1.70) mm (P = 0.39 vs. placebo group), and 1.59 (1.67) mm (P = 0.006, vs. placebo group), with corresponding angiographic binary restenosis of 79%, 67% (P = 0.38 vs. placebo group), and 54% (P = 0.08 vs. placebo group). The post-interventional primary patency rates at 3 months were 47%, 55% (P = 0.59 vs. placebo group), and 70% (P = 0.18 vs. placebo group) for placebo, 300 mg, and 600 mg groups. Our results demonstrated that intravenous 600 mg ascorbic acid was a feasible therapy and might attenuate restenosis after angioplasty; however, its effect on post-interventional primary patency was modest.

## Introduction

A well-functioning hemodialysis vascular access influences the morbidity and mortality of patients with end-stage renal disease. The most common cause of dialysis access dysfunction is stenosis of the outflow veins^1^. Percutaneous transluminal angioplasty (PTA) is widely used as a primary therapy for stenosis of dialysis access^2^. However, restenosis usually develops early after PTA, and the long-term durability of PTA is limited. Moreover, less than half of native accesses remained patent at 1 year, and the outcome is poorer for prosthetic accesses^2–4^. Restenosis usually requires repeated interventions, causing a large financial burden on the health care system. Various mechanical and pharmacological approaches have been developed to prevent restenosis^5–8^; however, the beneficial effects remain very limited and none of these approaches was recommended by guidelines^2^.

Uraemia is associated with increased oxidative stress and depletion of protective antioxidants, which are further complicated by dialysis^9^. Recent studies have demonstrated that oxidative stress and inflammatory cytokines are implicated in the stenosis of dialysis access^10,11^. In animal studies, antioxidants have been shown to prevent restenosis after angioplasty^12–14^. In humans, studies of coronary interventions also suggested a promising role of antioxidants in preventing restenosis^15–17^. Ascorbic acid (AA, vitamin C) is a potent antioxidant, and the plasma level of AA is generally lower in patients undergoing maintenance hemodialysis^18^. We and other investigators have previously shown that supplementation of AA in hemodialysis patients improved oxidative stress and inflammation, and corrected anemia^19–21^. Therefore, we conducted this feasibility study to test the hypothesis that AA could reduce the severity of restenosis after PTA and to evaluate the effect of two different doses (300 mg and 600 mg) of AA on restenosis after PTA.

## Materials and Methods

### Study design and study patients

This was a prospective, randomized, controlled, feasibility study designed to assess the feasibility of using two different doses of AA compared with the placebo in preventing restenosis of dialysis access after PTA. Patients undergoing maintenance hemodialysis were eligible for this study if they have had successful PTA at outflow veins of failing (but not failed) arteriovenous fistulas or grafts that have been created for at least 6 months. PTA was indicated based on the following criteria: (1) clinical signs, i.e., decreased thrill, development of collateral veins, limb swelling, and prolonged bleeding from puncture sites, suggesting vascular access dysfunction; (2) > 25% reduction of flow rate from baseline; (3) total access blood flow rate of <500 mL/min in arteriovenous fistulas and <600 mL/min in arteriovenous grafts, as assessed using the ultrasound dilution method (Transonic Flow-QC; Transonic Systems, Ithaca, NY, USA); and (4) increased venous pressure during dialysis, defined as dynamic venous pressure >150 mmHg in arteriovenous fistulas and >160 mmHg in arteriovenous grafts under a dialysis blood flow of 250 mL/min for three consecutive times. The exclusion criteria include patients who were (1) hospitalization for infection, heart failure, or acute coronary syndrome in the recent 3 months, (2) inability to comply with follow-up visits, and (3) use of AA or other antioxidant supplements before study enrollment. The study protocol was based on the Declaration of Helsinki (edition 6, revised 2000), approved by the institutional review board of National Taiwan University Hospital, Hsinchu Branch, and registered at ClinicalTrials.gov (number, NCT03524846; date, 30/04/2018). Informed consent was obtained from all participants.

### Randomization and study regimens

After the completion of the first hemodialysis session following successful index PTA, patients were randomly assigned into one of the three regimens: normal saline (placebo), 300 mg AA, and 600 mg AA. The doses of AA were chosen according to previous studies on anti-oxidant effect of AA in hemodialysis patients. After each dialysis session, 20 mL of 0.9% saline or sodium ascorbate at a dose of 300 mg or 600 mg was administered intravenously for 5 min, three times per week for 12 weeks. The treatment order was block-randomized using computer-generated numbers by the study nurse in our dialysis center. The patients, nephrologists, and interventionists were blinded to the study regiments.

### Angiography and angioplasty procedures

Diagnostic angiography (Advantx; GE Healthcare, Buc Cedex, France) was performed on a mid-week non-dialysis day. After diagnostic angiography, PTA was performed based on the National Kidney Foundation-Disease Outcomes Quality Initiative (NKF-DOQI) guidelines, i.e., only for patients with clinical indicators of dysfunction and a minimum of 50% diameter stenosis^2^. Stenosis was treated using a standard balloon angioplasty technique as previously described. High-pressure or cutting balloon was used only for lesions resistant to conventional balloon^6^. Drug-eluting balloon, stent or stent grafts were not used in this study. After the PTA procedure, antiplatelet therapy with aspirin or clopidogrel was administered for 3 days for all patients. Maintenance antiplatelet agents or other medications were added or continued according to the operators’ discretion or patients’ original indications.

### Quantitative angiographic analysis

A computer-based system (Digital DLX, GE Healthcare) was used for quantitative angiographic analysis. Measurement was performed by a physician who was blinded to the study information. The reference diameter (RD) was defined as an adjacent segment of normal vein located upstream to the target lesion. The means of the luminal diameter were used in patients who were undergoing PTA for more than one lesion. The absolute value of the minimal vessel diameter (MLD) was measured, and the degree of stenosis (DS) was reported as the maximum diameter reduction compared with the reference vessel diameter. The following parameters were derived: acute gain = MLD (DS) after PTA minus MLD (DS) before PTA and late loss = MLD (DS) after PTA minus MLD (DS) at follow-up.

### Clinical and angiographic follow-up

After successful PTA, all patients underwent prospective clinical follow-up evaluations for 12 weeks. Clinical follow-up surveillance included physical examination and dynamic venous pressure monitoring at each hemodialysis session and transonic examination of access blood flow rate immediately after the intervention and monthly until the end of 3-month period. The patients were referred for fistulography and PTA as appropriate if abnormal clinical or hemodynamic parameters were detected. All participants without clinical restenosis at the previously dilated area were scheduled for a follow-up angiography at the end of the study. The angiogram obtained before PTA was used as the follow-up angiogram if event-driven PTA was performed before the end of the study.

### Endpoints

The primary endpoint was the severity of restenosis, defined as the late loss of MLD or percentage stenosis at angiograms obtained before re-intervention or on follow-up angiograms obtained at the end of the study if no re-intervention was needed. Restenosis was also defined as a binary variable, i.e., >50% diameter stenosis at the target lesion. The post-interventional primary patency of the target lesion was defined as the interval between intervention and the next access thrombosis or repeated intervention at the previously treated area within 3 months. The post-interventional primary patency of the access circuit was defined as the interval between intervention and the next thrombosis or repeated intervention at anywhere from the arteriovenous junction to the central vein of the access circuit. These definitions were in accordance with the guidelines of the Society of Interventional Radiology^22^.

### Statistical analysis

A previous study using AA to prevent restenosis of coronary arteries had shown a mean 30% reduction in late loss and 25% reduction in restenosis severity. We calculated that 228 patients were needed to detect a 30% reduction of late loss with a power and two-tailed significance level of 0.80 and 0.05, respectively. Because the number of patients needed to detect a difference in restenosis severity was more than the patients in our dialysis unit, we designed a feasibility study to explore the effect of AA and the adequate dose for hemodialysis vascular accesses. We enrolled 30 patients after PTA in each of the placebo, 300 mg AA, and 600 mg AA groups. A post-hoc power calculation will be performed based on the data from this study. Continuous variables were expressed as means (standard deviation, SD) for normally distributed data or medians (interquartile range, IQR) for non-normally distributed data, and proportions for categorical data. Differences between the AA and the placebo groups were compared by using the *t*-test and Mann-Whitney test for normally and non-normally distributed data. Categorical data were compared using the chi-square test with Yates’ correction and Fisher’s exact test as appropriate. Post-interventional patency was analyzed using the Kaplan–Meier method, and between-group comparisons were using the long-rank test. A two-tailed P value of <0.05 was considered statistically significant. Statistical analysis was conducted using SPSS, version 20.0 (SPSS Inc., Chicago, IL, USA).

## Results

**Figure 1:**
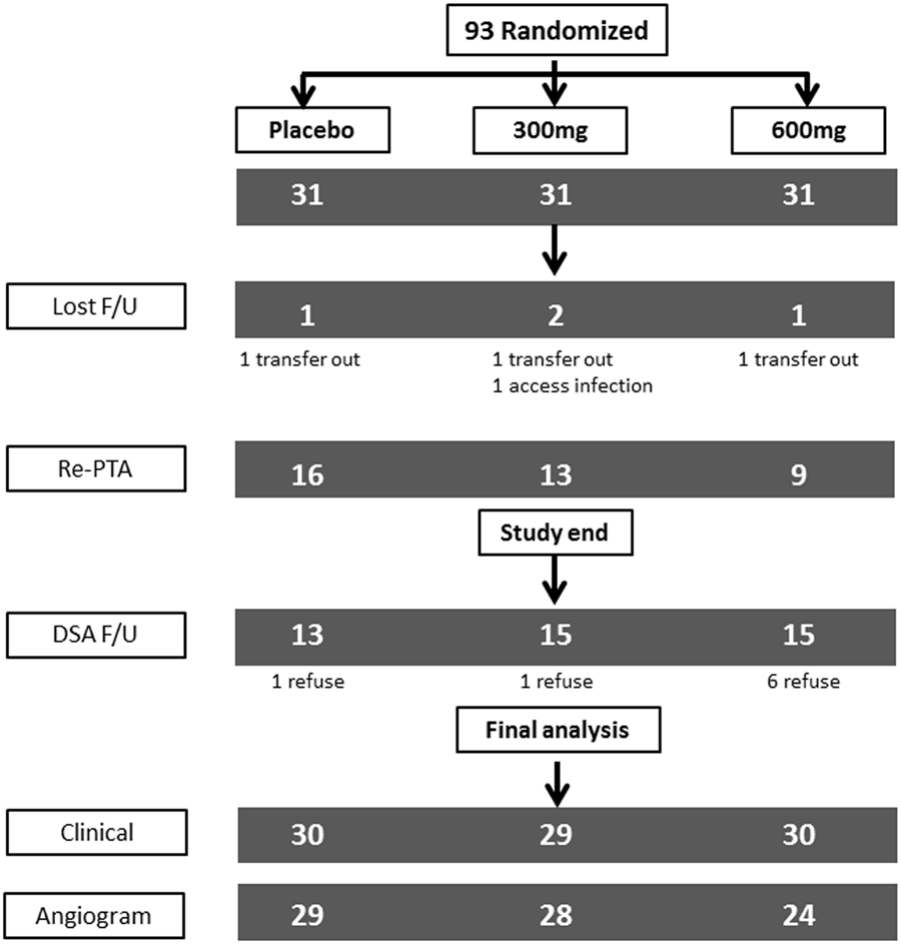
Flow of study participants.

Ninety-three patients were randomized into three groups from April to October, 2011, with 31 patients were in each group (Fig. 1). Their mean age was 65 years (SD, 12 years), and the median dialysis vintage was 50 months (IQR, 22–74 months). Table 1 shows the demographic and clinical data, and medications of the three groups at baseline. No significant difference was found in the baseline parameters among the three groups. The median shunt vintage was 41 months (IQR, 21–65 months); 64 (68%) and 81 (86%) were prosthetic accesses and restenotic lesions, respectively. Table 2 shows the characteristics of dialysis access, lesions, and procedures of the three groups. No significant difference was found in the baseline vascular and procedural factors among the three groups.

**Table 1:** Baseline characteristics.

Factors	Placebo	Ascorbic acid		*P vs. placebo*	
		300 mg	600 mg	300 mg	600 mg
	(N = 31)	(N = 31)	(N = 31)		
**Demographic factors**					
Age (yr)	67 (13)	64 (12)	65 (13)	0.68	0.78
men/women	11/20	17/14	14/17	0.20	0.61
HD duration (mo)	52 (27, 67)	31 (17, 54)	48 (22, 75)	0.18	0.83
**Risk factors**					
Hypertension	25 (81%)	25 (81%)	25 (81%)	0.99	0.99
Diabetes	15 (48%)	20 (65%)	12 (39%)	0.31	0.61
Hyperlipidemia	14 (45%)	11 (36%)	9 (29%)	0.61	0.29
Current smoker	8 (26%)	9 (29%)	7 (23%)	0.73	0.95
Coronary artery disease	19 (61%)	14 (45%)	16 (52%)	0.31	0.61
**Presentation**					
Inadequate flow	9 (29%)	11 (36%)	12 (39%)	0.79	0.59
High pressure	22 (71%)	20 (64%)	19 (61%)	0.79	0.59
**Biochemistry profile**					
Cholesterol (mg/dL)	163 (36)	170 (62)	162 (38)	0.88	0.99
Albumin (g/dL)	3.6 (0.4)	3.7 (0.4)	3.6 (0.3)	0.48	0.89
Hemoglobin (g/dL)	10.9 (1.6)	10.8 (2.1)	10.8 (1.5)	0.96	0.96
Calcium (mg/dL)	9.4 (0.8)	9.5 (1.1)	9.4 (0.7)	0.94	0.98
Phosphate (mg/dL)	4.7 (1.5)	4.3 (1.6)	4.8 (1.6)	0.53	0.99
Kt/V	1.4 (0.2)	1.3 (0.2)	1.3 (0.2)	0.33	0.36
**Post-angioplasty maintenance medications**					
Anti-platelets	11 (36%)	13 (42%)	13 (42%)	0.79	0.78
RAS inhibitors	13 (42%)	15 (48%)	12 (39%)	0.80	0.99
Statin	13 (42%)	8 (26%)	10 (32%)	0.28	0.60

HD, hemodialysis; Kt/V, urea clearance; RAS, renin-angiotensin system.

Hypertension: systolic blood pressure $\underline{\underline{ > }}$140 mmHg or diastolic blood pressure $\underline{\underline{ > }}$90 mmHg or use of anti-hypertensive drugs; Diabetes, fasting glucose $\underline{\underline{ > }}$126 mg/dl or using anti-diabetic drugs; hyperlipidemia, total cholesterol $\underline{\underline{ > }}$240 mg/dl; coronary artery disease, evidence of myocardial ischemia by non-invasive or invasive tests.

**Table 2:** Baseline procedure and lesion characteristics.

Factors	Placebo	Ascorbic acid		*P vs. placebo*	
		300 mg	600 mg	300 mg	600 mg
	(N = 31)	(N = 31)	(N = 31)		
**Access**					
Shunt age (mo)	45 (24, 53)	29 (17, 43)	59 (19, 72)	0.12	0.51
Native access	11 (36%)	10 (32%)	8 (26%)	0.99	0.58
Upper arm access	5 (16%)	5 (16%)	3 (10%)	0.99	0.71
Right arm access	5 (16%)	3 (10%)	5 (17%)	0.71	0.99
**Lesion**					
RD (cm)	0.68 (1.32)	0.67 (1.24)	0.68 (0.83)	0.97	0.99
Length (cm)	2.0 (0.7)	2.1 (0.9)	1.9 (0.8)	0.95	0.79
Angulation >45°	2 (7%)	3 (10%)	1 (3%)	0.99	0.99
Multiple lesions	7 (23%)	4 (13%)	6 (19%)	0.51	0.99
Restenotic lesions	28 (90%)	29 (94%)	24 (77%)	0.99	0.30
Locations				0.82	0.67
Arterial anastomosis	2	3	4		
Venous anastomosis	11	6	9		
Proximal vein	7	9	7		
Cephalic vein	7	6	7		
Basilic vein	8	11	7		
**Procedure**					
Oversizing >10%	8 (26%)	11 (36%)	9 (29%)	0.58	0.99
High pressure >14 atm	8 (26%)	6 (19%)	7 (23%)	0.76	0.99
Cutting balloon	1 (3%)	0 (0%)	0 (0%)	0.99	0.99
**Complications**					
Vessel rupture	1 (3%)	3 (10%)	1 (3%)	0.61	0.99
Dissection	0 (0%)	1 (3%)	0 (0%)	0.99	0.99

RD, reference diameter.

### Clinical follow-up

**Figure 2:**
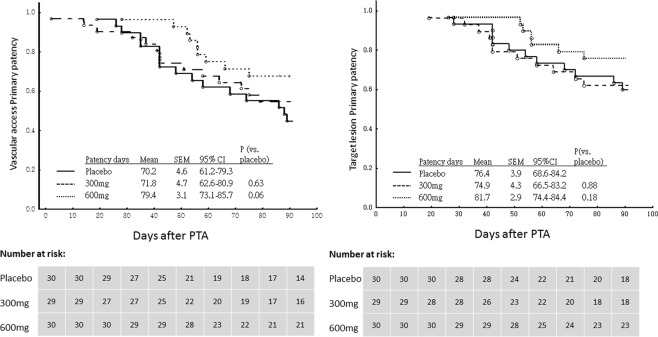
Kaplan–Meier plots of post-interventional primary patency of access circuit (left) and target lesion (right) within a 3-month follow-up period after angioplasty, stratified by placebo, 300 mg, and 600 mg ascorbic acid groups.

No death occurred during the study period. Three patients, one in each group, were lost to follow-up owing to transfer to other hemodialysis centers. One patient in the 300 mg AA group had an infected access, which was removed by the end of the study. These four patients discontinued the study medications prematurely and were also excluded from the final analysis. All the remaining 89 patients were adherent to the study regimens and completed the clinical follow-up. During the 3-month follow-up period, 38 (43%) patients underwent re-interventions. Table 3 shows the indications for re-interventions. The incidence rate of re-intervention was 53%, 45%, and 30% for the placebo, 300 mg AA (P = 0.59 vs. placebo group), and 600 mg AA (P = 0.18 vs. placebo group) groups, respectively. Figure 2 shows the post-interventional primary patency of the access circuit according to the Kaplan–Meier plots. Stenosis occurred in 34 patients (37%) and was the most common cause of re-intervention. Of the 34 stenosis, 30 occurred in the previously dilated venous segment. The target lesion restenosis rate was 40%, 37%, and 23% for the placebo, 300 mg AA (P = 0.99 vs. placebo group), and 600 mg AA (P = 0.27 vs. placebo group) groups, respectively. The post-interventional primary patency of the target lesion is shown in the Kaplan-Meier plots in Fig. 2. The number of access thrombosis and access failure was relatively small: six patients had thrombosis, and one patient had access loss due to infection. None of the patients discontinued the study medications. At the end of the study, the control, 300 mg AA, and 600 mg AA groups had five, two, and three hospitalizations, respectively. All hospitalizations were not related to the adverse effects of the study medications.

**Table 3:** Clinical events during the 3-month follow-up.

Factors	Placebo	Ascorbic acid		*P vs. placebo*	
		300 mg	600 mg	300 mg	600 mg
	(N = 30)	(N = 29)	(N = 30)		
**Re-intervention**	16 (53%)	13 (45%)	9 (30%)	0.59	0.18
Stenosis					
Target lesion	12 (40%)	11 (37%)	7 (23%)	0.99	0.27
Access circuit	15 (50%)	12 (41%)	7 (23%)	0.30	0.06
Thrombosis	1 (3%)	3 (10%)	2 (7%)	0.61	0.99
**Access failure**	0 (0%)	1 (3%)	0 (0%)	0.49	0.99
**Hospitalization**					
Cardiovascular	1 (3%)	1 (3%)	2 (7%)	0.99	0.99
Non-cardiovascular	4 (13%)	1 (3%)	1 (3%)	0.20	0.35
**Death**	0 (0%)	0 (0%)	0 (0%)	0.99	0.99

Target lesion: stenosis at previous dilated area.

Access circuit: Stenosis at anywhere from the arteriovenous junction to the central vein.

### Angiographic follow-up

**Figure 3:**
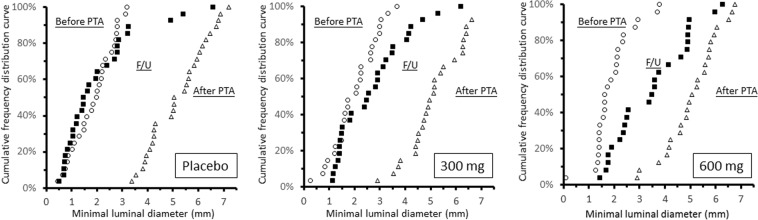
Cumulative frequency distribution curves of minimal luminal diameter (MLD) before percutaneous transluminal angioplasty (PTA), after PTA, and at follow-up (F/U) angiograms stratified by placebo, 300 mg, and 600 mg ascorbic acid groups.

**Figure 4:**
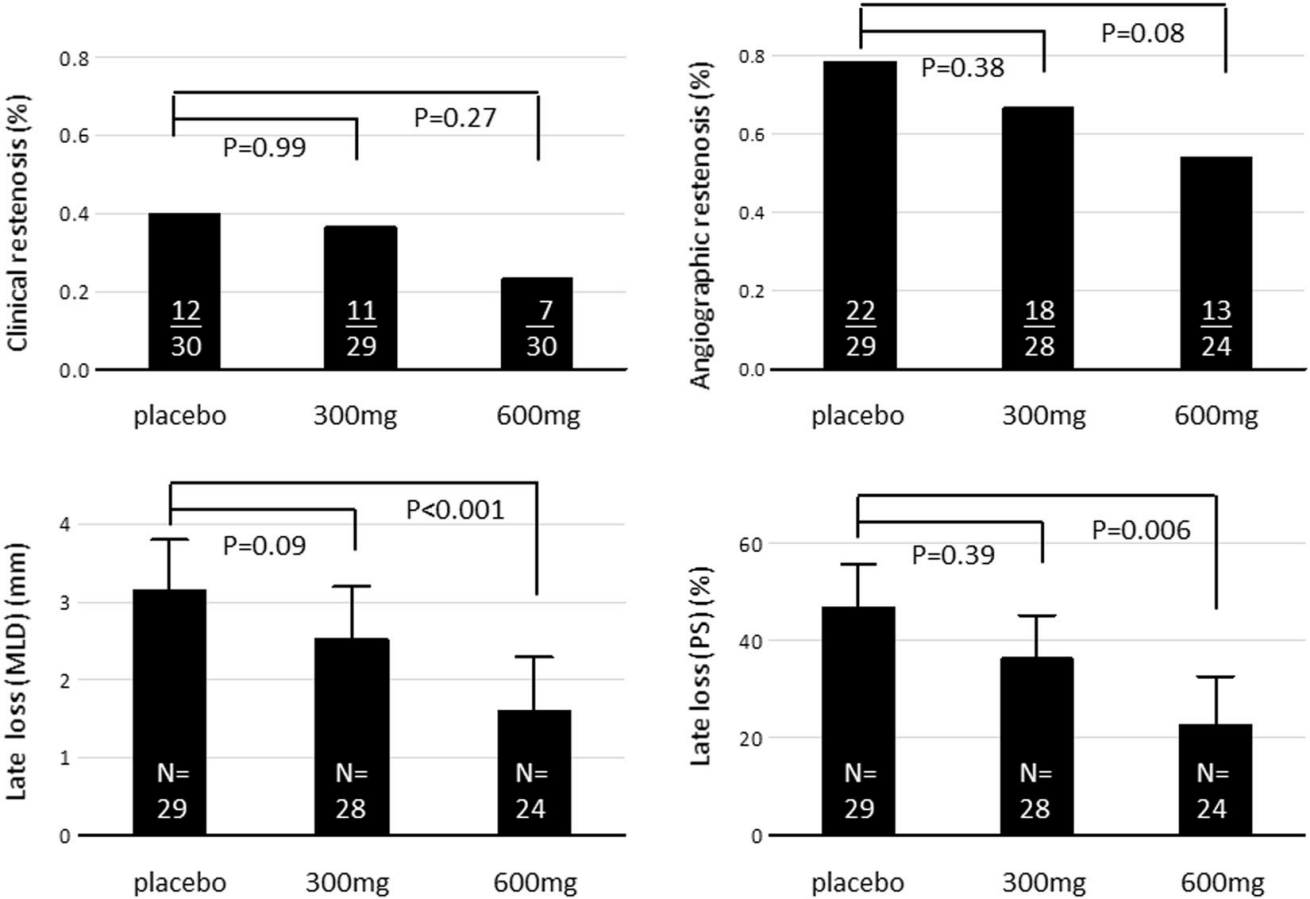
Comparison of clinical restenosis rate, angiographic binary restenosis rate, late loss by minimal luminal diameter (MLD), and late loss by diameter stenosis (DS) between placebo, 300 mg, and 600 mg ascorbic acid groups.

Angiographic follow-up included angiographies before the target-lesion PTA in 38 patients with symptomatic restenosis and angiographies at the end of the study in another 43 asymptomatic patients. Eight patients refused angiographic follow-up at the end of the study. Table 4 summarizes the results of quantitative angiographic analysis of the 81 patients with angiographic follow-up. The late loss of MLD among these patients was 3.15 mm (SD, 1.68 mm), 2.52 mm (1.70 mm), and 1.59 mm (1.67 mm) in the placebo, 300 mg AA (p = 0.39 vs. placebo), and 600 mg AA (P = 0.006 vs. placebo) groups, respectively. Figure 3 shows the cumulative frequency curves of MLD before PTA, after PTA, and at follow-up of each study group. Angiographic target lesion binary restenosis was found in 22 (79%), 18 (67%), and 13 patients (54%) of the placebo, 300 mg AA (P = 0.38 vs. placebo), and 600 mg AA (P = 0.08 vs. placebo group) groups, respectively. (Fig. 4).

**Table 4:** Quantitative angiographic data at baseline and follow-up.

Factors	Placebo	Ascorbic acid		*P vs. placebo*	
		300 mg	600 mg	300 mg	600 mg
	(N = 29)	(N = 28)	(N = 24)		
**MLD (mm)**					
Before PTA	1.89 (0.80)	1.99 (0.86)	1.92 (0.80)	0.88	0.99
After PTA	5.22 (1.12)	5.28 (1.25)	5.08 (1.04)	0.98	0.90
Follow-up	2.08 (1.51)	2.76 (1.39)	3.49 (1.44)	0.23	0.003
Acute gain	3.34 (1.23)	3.28 (1.46)	3.17 (1.29)	0.98	0.90
Late loss	3.15 (1.68)	2.52 (1.70)	1.59 (1.67)	0.39	0.006
**Stenosis (%)**					
Before PTA	72 (11)	70 (12)	71 (12)	0.81	0.99
After PTA	23 (10)	21 (11)	25 (13)	0.91	0.74
Follow-up	70 (19)	58 (23)	48 (14)	0.13	0.002
Acute gain	49 (15)	48 (18)	46 (17)	0.85	0.51
Late loss	47 (23)	36 (22)	23 (24)	0.09	<0.001
Binary restenosis	22 (79%)	18 (67%)	13 (54%)	0.38	0.08

Binary restenosis: more than 50% diameter stenosis; MLD, minimal luminal diameter; PTA, percutaneous transluminal angioplasty.

Minimal luminal diameter and stenosis percentage between groups was compared by t test; binary stenosis between groups was compared by chi-square test.

## Discussion

To date, no pharmacological strategy has been proved to prevent restenosis after PTA of dialysis vascular access. Data from our study provided the first evidence showing that AA therapy may attenuate the severity of restenosis. Compared with placebo, administration of 600 mg AA after each dialysis session for 3 months decreased late luminal loss by 50%. Furthermore, the restenosis rate of the target lesion also decreased from 41% to 24%. According to the post-hoc statistical power calculation, our number of patients were not powered to detect a difference in primary patency rate of the access circuit (power = 0.59) or the binary restenosis of target lesions (power = 0.49). Nonetheless, a significant decrease in the severity or restenosis was obtained in the 600 mg AA group, with a statistical power of 0.92. Our study provided a proof of feasibility that intravenous AA could be used after PTA and was potentially effective in preventing restenosis for dialysis vascular access. Furthermore, the preliminary data also provided a basis for power calculation in future pivotal trials. According to the difference in our data, a sample size of 240 patients in each of placebo and 600 mg AA group will have a power of 0.80 to detect a benefit on patency of dialysis access circuit.

In this study, both clinical and angiographic effects were assessed to delineate the benefit of AA. According to the quantitative angiographic analysis, the 600 mg group had a 49% reduction of late loss by luminal diameter (1.59 mm vs. 3.15 mm) and a 51% reduction of late loss by percentage stenosis (23% vs. 47%) compared with the placebo group. The attenuating effect was less prominent in the 300 mg group, and either the improvement of late loss by diameter (20%) or percentage stenosis (23%) did not achieve statistical significance. Previous studies found a similar efficacy of antioxidants against post-angioplasty restenosis after coronary interventions. In the study by DeMaio *et al*., 1200 IU of vitamin E per day showed a trend toward reduction of restenosis for patients undergoing coronary angioplasty^23^. In the study by Tardif *et al*., patients administered with probucol had a 68% reduction of late luminal loss^15^. In another study by Yokoi *et al*., probucol reduced the late loss of luminal diameter and percentage stenosis by 38% and 61%, respectively^17^. In another study of coronary angioplasty, administration of AA 500 mg per day resulted in a 32% and 43% reduction in the late luminal loss and binary restenosis rate, respectively^16^. The attenuating effect in our study was consistent with that in previous reports, supporting a favorable effect of antioxidants in inhibiting intimal hyperplasia, both for arterial and venous diseases.

Despite the attenuating effect on late luminal loss, the reduction in angiographic restenosis did not translate into reduction in re-interventions. Both symptomatic restenosis of the whole access circuit (38% vs. 57%) or target lesions (24% vs. 41%) were lower in the 600 mg AA group than in the placebo group. Nonetheless, the reduction in restenosis rate did not reach statistical significance. The patency rate in our study was lower than that in previous reports with mostly retrospectively ascertained data. The patency rate has been well known to be lower when it was prospectively assessed than retrospectively ascertained^5,24,25^. Furthermore, 68% of our patients had prosthetic access, and 86% had restenotic lesions. The unfavorable characteristics of the accesses may account for the low patency rate in our study. Finally, our study used a formal angiography follow-up was used at the end of the study for asymptomatic patients. Angiographic restenosis was 54% and 79% in the 600 mg AA and placebo groups, respectively, which was higher than and similar to that in other studies with angiographic follow-up, respectively^5^.

A previous study had shown a reduction of target lesion restenosis rate from 39% to 22% after the administration of AA for 4 months^16^. The small sample size of our study may limit our ability to find a clinically significant benefit. Furthermore, as demonstrated in our study, the restenosis rate of dialysis access was very fast and extensive^4,5^. In our study, up to 67% of the patients with experienced symptomatic restenosis within 3 months, and the restenosis rate may be up to 79% when a routine angiographic follow-up was performed. Such a rapid speed of restenosis and a high proportion of restenosis may offset the potential benefit of AA. Furthermore, various causes may be responsible for restenosis of dialysis access, in addition to inflammation and oxidative stress^9^. Antioxidant therapy could reduce oxidative stress and inflammation but did not seem to be helpful in other pathogenic mechanisms, such as hemodynamic stress, hemostasis, and needle injury.

Hemodialysis patients have been well known to have a lower plasma AA level than the normal population^18^. Many literatures documented 100–200 mg/day oral AA or 300–500 mg thrice weekly intravenous AA are sufficient and safe for hemodialysis patients^26^. For using AA to decrease oxidative stress, the optimal dose, route, and duration of administration are controversial. In studies showing that AA decreased oxidative stress, 250 mg/day orally for 12 weeks, 1 g/day orally for 1 year, intravenous doses of 300 mg/day intravenously for 8 weeks, and 1 g/day intravenously for 2 months had been administered^19,27–29^. Some studies showed no change in oxidative stress when a single intravenous dose or a daily dose of 250 mg for 4–12 weeks was used^29–32^. Because of these controversial results in previous studies, we tested two doses of AA: 300 mg and 600 mg AA. AA was administered intravenously immediately after each dialysis session to enhance compliance. Although no statistically significant difference in binary stenosis was observed, a dose-dependent decrease in the extent of restenosis, assessed according to late luminal loss, was found between 600 mg and 300 mg compared with the placebo group. Further studies may be warranted to determine if a higher dose or a pre-treatment strategy could achieve a prominent effect.

There are several mechanisms by which AA treatment could attenuate restenosis after PTA. Damaged endothelium, activated platelets, and neutrophils at the angioplasty site will generate reactive intermediates. These oxidative metabolites can induce endothelial dysfunction and activate macrophage, which in turn, can release several growth factors that promote tissue proliferation^33^. In hemodialysis patients, uraemia and dialysis are associated with increased oxidative stress that causes activation of inflammation, release of free radicals, and depletion of protective antioxidant. Recent studies have shown that pro-inflammatory chemokines and oxidative stress markers are implicated in venous intimal hyperplasia of dialysis vascular access^10,11,34^. Experimental studies also demonstrated that AA reduced oxidant stress levels, improved endothelial function, and decrease expression of vascular adhesion molecules, growth factors, and chemokines that may play an important role in the neointimal formation^35–37^. In addition, AA may also be able to change the ratio of prostacyclin to thromboxane and to reduce platelet aggregation and vasoconstriction^38^. These beneficial effects at the cellular levels have been translated in animal models. One study in pig model reported that a combination of vitamins C and E decreased intimal thickening after angioplasty. In another study in rats, vitamin C intake decreased the degree of atherosclerosis formation. In humans, a combination therapy of vitamins C and E decreased the intimal index in patients who underwent cardiac transplantation. Another study in non-uremic patients showed a possible effect of AA in attenuating restenosis after angioplasty of coronary arteries. Our study provided the first evidence showing that AA also attenuated intimal hyperplasia at outflow veins of dialysis access.

This study has limitations. Dosages of AA as high as 500 to 1000 mg/day for 3 or >3 weeks may significantly induce increased plasma oxalate levels^39,40^. Although all patients tolerated the regimens well, we did not measure the plasma level of oxalate before and after study. Although the variation of vitamin C from dietary intake or multivitamin supplementation was small, it was not controlled in this study and might dull the effect of AA. Early re-intervention may be secondary to elastic recoil, not necessarily intimal hyperplasia. Nonetheless, the interference of recoil should be minimized by subtraction of post-PTA stenosis in the calculation of late loss. Most of these lesions were restenotic lesions and the effect of AA on primary stenosis still needs to be determined. We did not measure the plasma AA level because plasma AA levels and pharmacokinetic data of intravenous AA administration have been reported in previous literatures^19,41,42^. The drop-out rate in the 600 mg AA group was higher than that in other groups because fewer patients in this group experienced recurrent vascular access dysfunction. Finally, the study cohort is composed of two third of patients on arteriovenous grafts. A high proportion of graft accesses may limit the applicability of this study because native fistulas are more commonly used in hemodialysis patients.

In conclusion, our results showed that intravenous administration of AA at a dose of 600 mg three times per week attenuated the severity of restenosis after PTA. Currently, devices, such as stent graft and drug-eluting balloon, had been used or under investigation to prolong the durability of PTA. Nonetheless, the absolute increase in the patency attributable to the use of expensive devices was still limited. Although a clinically significant improvement in patency could not be achieved, therapy with AA was safe and inexpensive. A multi-disciplinary strategy to prolong patency by combining AA therapy with use of mechanical devices deserved further investigation.
